# Three Dimensional Bioprinting for Hepatic Tissue Engineering: From *In Vitro* Models to Clinical Applications

**DOI:** 10.1007/s13770-023-00576-3

**Published:** 2023-10-26

**Authors:** Meghana Kasturi, Vidhi Mathur, Mrunmayi Gadre, Varadharajan Srinivasan, Kirthanashri S. Vasanthan

**Affiliations:** 1 Manipal Centre for Biotherapeutics Research, Manipal Academy of Higher Education, Manipal, Karnataka 576104 India; 2 Department of Civil Engineering, Manipal Institute of Technology, Manipal Academy of Higher Education, Manipal, Karnataka 576104 India

**Keywords:** 3D bioprinting, Bioink, Liver regeneration, Disease model, Drug screening

## Abstract

Fabrication of functional organs is the holy grail of tissue engineering and the possibilities of repairing a partial or complete liver to treat chronic liver disorders are discussed in this review. Liver is the largest gland in the human body and plays a responsible role in majority of metabolic function and processes. Chronic liver disease is one of the leading causes of death globally and the current treatment strategy of organ transplantation holds its own demerits. Hence there is a need to develop an *in vitro* liver model that mimics the native microenvironment. The developed model should be a reliable to understand the pathogenesis, screen drugs and assist to repair and replace the damaged liver. The three-dimensional bioprinting is a promising technology that recreates *in vivo* alike *in vitro* model for transplantation, which is the goal of tissue engineers. The technology has great potential due to its precise control and its ability to homogeneously distribute cells on all layers in a complex structure. This review gives an overview of liver tissue engineering with a special focus on 3D bioprinting and bioinks for liver disease modelling and drug screening.

## Introduction

Liver is the largest gland in the human body and is responsible for metabolic functions including production of bile, albumin and cholesterol, regulation of amino acids, filters blood, resists infections and processes glucose. Liver is only organ in the human body that can efficiently regenerate, and hence any damage to the organ leads to various disorders and complications. However, extensive use of drugs can damage the hepatocytes beyond repair and limit its regeneration capabilities leading to liver failure, which in turn gives raise to complications such as neurological impairment, renal dysfunction, and metabolic abnormalities [1]. Chronic liver diseases like fibrosis, cirrhosis, chronic viral hepatitis, fatty liver disease etc. affects people of all age group and race [2] and are on raise globally due to change in lifestyle and contribute to the global healthcare burden (mortality and morbidity) with Cirrhosis, end stage of liver fibrosis ranking the eleventh leading cause of death in the world. According to the latest statistical data, liver diseases are the eighth leading cause of death in India (World Health Organization 2020) [3]. The World Health Organization (WHO) recently estimated that 296 million people were living with chronic hepatitis B infection. The hepatitis B infection caused an estimated 820 000 deaths, mostly from cirrhosis and hepatocellular carcinoma. Globally, there were 36,000 fatal cases due to acute hepatitis B. Hepatitis B virus (HBV) mortality is high but will decline since vaccines for the same are now available [4]. Viral hepatitis has been found to be a leading cause for chronic liver diseases all around the world and two main causes of viral hepatitis is been Alcoholic liver disease (ALD) and Non-alcoholic fatty liver disease (NAFD) [5]. Table 1 summarises the prevalence of liver diseases in India. The current medical intervention available for liver failure is the partial or full liver transplantation. However, organ transplantation has its own shortcomings such as availability of healthy donors, immune response after transplantation and rate of engraftment success. Other tissue engineering options available as treatment includes bioartificial liver system, hepatocyte transplantation, cell therapy approaches with certain shortcomings [6]. Tissue engineering strategies offer alternative solution by fabricating *in vitro* tissues to repair or replace the damaged part of the liver.

**Table 1:** Frequency of different liver diseases in India (copyright obtained from [6])

Liver diseases	Causes	Prevalence (%)	Contribution to mortality (%)
Liver cancer	HBV	46.8	40–60
	HCV	14.8	10–20
	NAFLD/NASH	4.6–19	5–10
	Alcohol	9.6	15–20
Chronic liver disease	HBV	17.6–47.9	30–60
	HCV	5.2–44.9	10–22
	NAFLD/NASH	2.6–43.6	10–15
	Alcohol	10.9–31.9	20–25
	Others*	9.7–23.2	5–10
Acute liver disease	HAV	1.7–33	5–6.3
	HEV	30–50	30–40
	HBV	13.9–27.6	55–60
	Non-A-E virus	14.6–43.9	0.5–2
	Drugs	0–15	–

Others* include autoimmune hepatitis and other autoimmune liver diseases, Wilson’s disease, and unknown causes

The 3D printing has driven innovations in many areas and has changed the paradigm of “design for manufacturing” to “design for function”. This technology was first introduced by Charles Hull in 1986 where a material was printed in a layer-by-layer fashion and was termed as stereolithography. Since then, the models have been developed in different shapes, sizes and colour for the manufacturing industry using resin, plastic, metal etc. [7]. 3D bioprinting is an additive manufacturing process where the cells, proteins and biomaterials are printed into desired structures. 3D bioprinting helped in automating and scaling up the entire process and provides precision and reproducibility. A lot of research is focused on improving bioinks for shape fidelity, elasticity, cell viability, to mimic the desired native tissue. These innovations have led to fabrication of functional tissues with 3D bioprinting technology while the futuristic approach is their clinical translation [8]. 3D printed patient specific liver models are on the rise as they show great potential in modelling diseases accurately and reduce the time and money for drug screening process [9].

**Fig. 1:**
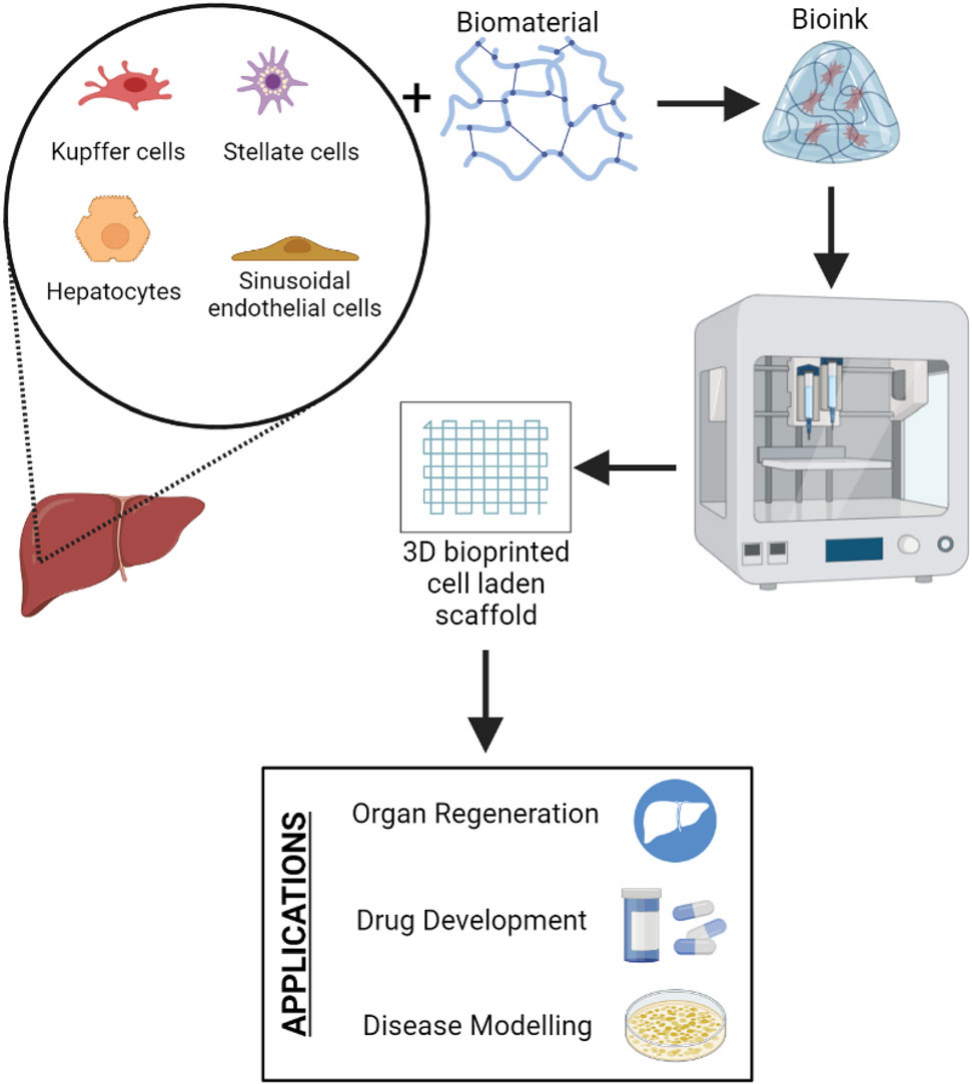
Overview of 3D bioprinting liver

In this review, we focus on 3D bioprinting based liver models for liver regeneration, drug screening, disease models, surgical applications, clinical translations, and commercially available products. Figure 1 represents the overview of 3D bioprinting liver and its applications. Furthermore, a brief introduction on various tissue engineering techniques for *in vitro* liver models is given in this review which majorly focuses on 3D bioprinting. 3D bioprinting is a promising technique for the development of biocompatible structures that mimic the native organ’s architecture. However, liver tissue engineering has many challenges since the liver is a highly vascular organ. Hence, creating a similar microenvironment could be challenging in order to generate a functional liver tissue. The review also discusses the current challenges and potential solutions along with available cell sources, and suitable bioinks and bioprinting strategies.

## Liver architecture and functions

**Fig. 2:**
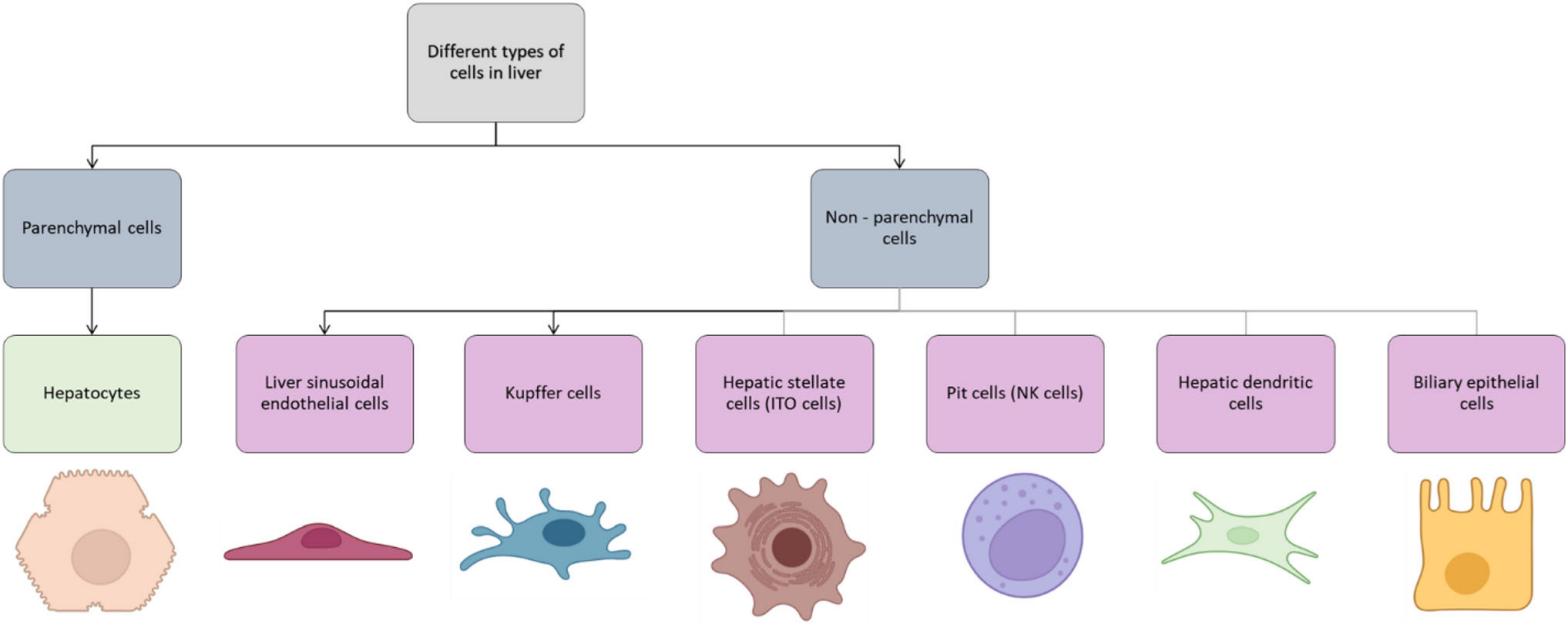
Liver cell types [11]

**Fig. 3:**
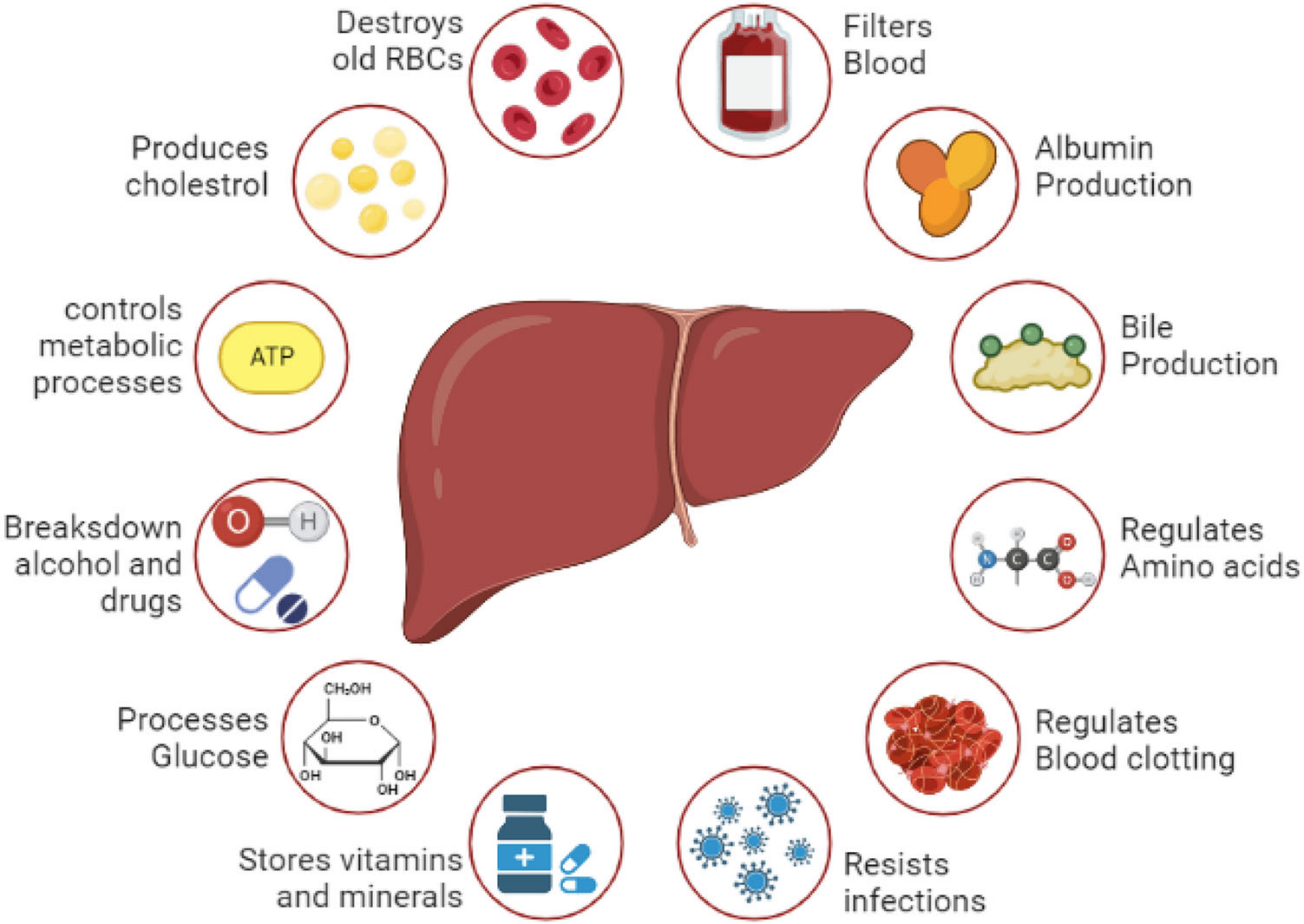
Functions of liver

**Fig. 4:**
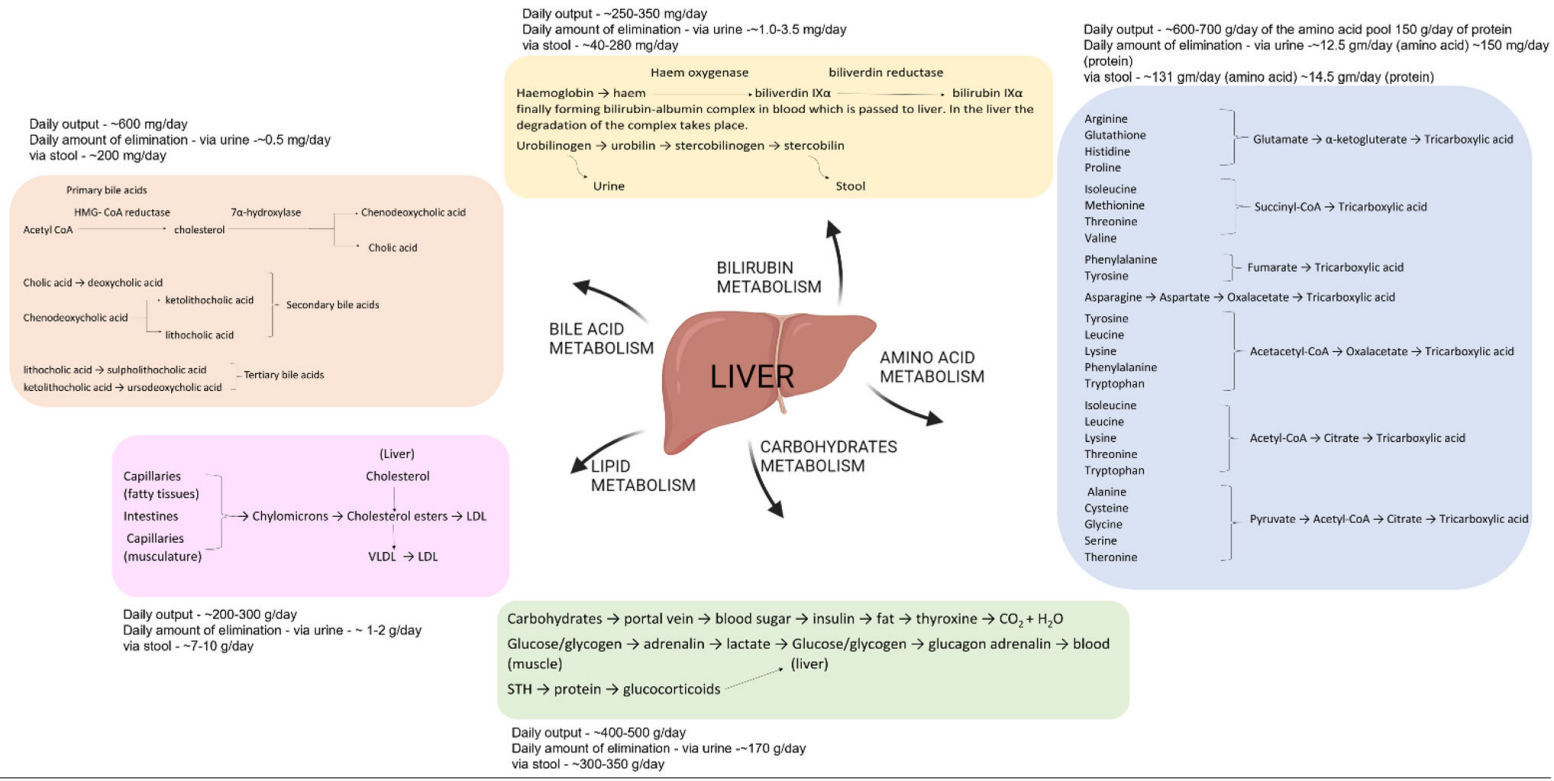
Metabolic pathways of liver

The human liver makes up around 1/50th of the total body weight and histological slices of the liver reveal uniform landscape of cells and tissue architecture. There are four lobes present in the adult human liver, namely right lobe, left lobe, caudate and quadrate, which are made up of different cell types in which the hepatocytes make up 80% of the liver cell population [10]. Figure 2 illustrates the different cell populations present in a human liver. The five main systems that make up the liver are—(a) intrahepatic vascular system, (b) stroma, (c) sinusoidal cells, (d) hepatocytes and biliary epithelial cells, and (e) Hepatic lobules and acinus; each of them contributing to total functioning of the liver. The liver is known to have multiple functions that is associated with digestion of food, detoxifying the toxins, haemostasis, balancing the fluids and electrolytes (Fig. 3). These activities are grouped under metabolism, bile secretion and detoxification [12]. The collective functioning of the liver cells helps in understanding the liver functioning mechanism as an organ. The key to the liver's proper operation rests on the ongoing maintenance of a variety of biochemical activities, including the many metabolic processes taking place in the liver's hepatocytes and sinusoidal cells. It is challenging to fully understand all the functions of the liver because of the multiple connections between various metabolic pathways and functional activities. Some of these pathways are represented in Fig. 4. In addition to assisting cellular absorption and excretory activities, metabolic processes entail several distinct and contrasting biochemical paths to enable the synthesis of breakdown and the activation or deactivation of substances [13]. The liver's additional crucial purpose is to supply the right amounts of solutes for the proper operation of all the different organs including the essential organs—the brain, heart, and kidneys. A regulated metabolic system is needed to maintain the complex web of biochemical processes that take place inside the liver cell. Different regulatory metabolism take place at different levels which includes molecular, cellular and organ levels [14–16]. The liver capacity for normal functioning fails following the cell death where the damage exceeds the capacity of regeneration and repair. To limit the liver injury, it is an endogenous defence mechanism that operates on cells by recruiting them before it is in an irreversible stage of damage and during this process the cells are condemned to die via apoptosis rather than necrosis. A huge number of factors are involved in the regenerative activities of the cells at various intracellular events which includes extracellular signalling, auxiliary mitogenic signals and complex mitogenic pathways which are summarized in Table 2.

**Table 2:** Factors affecting liver regeneration

Sl. no.	Factors of liver regeneration	Effects of the factors on the liver	Referencess
1	Factors from extra-cellular signalling		
	A. HGF	The liver ECM contains HGF and is triggered post partial hepatectomy and ECM remodelling, increasing tenfold normal plasma concentrations in less than an hour	[17]
		HGF mRNA starts to grow in first three hours and peaks at 15 h, causing the synthesis of new HGF	[18]
		An increase in the circulation of noradrenaline, induces the formation of HGF in MRC-5 cell line	[19]
		HSCs make HGF in the healthy liver and throughout the initial phases of liver regeneration, and is controlled by p75NTR61. HGF migrates from endothelial cells from bone marrow during regeneration	[20]
	B. HB-EGF	LSECs production of HB-EGF keeps HSCs dormant but is activated by TGFα	[21]
		It is unclear; however hepatocyte proliferation has been observed in hepatocytes due to HB-EGF activation	[22]
		Bblocking of either EGFR or MET signalling slowed but did not totally stop liver regeneration. However, blocking both signalling pathways at the same time completely stopped liver regeneration	[23]
		On day fourteen each mice from their study died from numerous metabolic abnormalities, due to supression of HB-HGF leading to disorder of liver metabolism	[24]
		The combined ablation of MET and EGFR signalling eliminates hepatocyte transdifferentiation and liver enlargement induced by CAR in response to xenobiotic mitogens	[25, 26]
	D. β-Klotho	FGFR4 or β-Klotho knockdown increases mortality, which is a FGFR4-coreceptor	[27]
		Bile communicates with FGFR4-Klotho to suppress CYP7A1. Hepatic bile acid levels significantly rise in FGF15-knockout mice and mortality increases	[28]
2	Factors from Auxiliary mitogenic signals		
	A. TNFR	Mice lacking TNFR1 or TNFR2 exhibit delay in liver regeneration and reduced in activating NF-κB	[29–31]
		After partial hepatectomy, blood levels of IL-6 is generated by liver cells, also rise quickly	[32, 33]
		In IL-6-knockout mice, STAT3 activation in hepatocytes is delayed	[34, 35]
		In absence of TNFα, both NF-κB and STAT3 are activated in culture medium when supplemented with HGF and EGF	[36]
		rat's level of noradrenaline is generated HSCs, rise at the same pace as HGF	[37]
	B. Noradrenaline	Noradrenaline increases EGFR and MET, transactivates STAT3, and inhibits TGF-1 ability to stop hepatocytes from migrating	[38–40]
		Noradrenaline increases the synthesis of EGF and HGF by Brunner glands and fibroblasts, respectively	[41–43]
	C. Bile acids	Bile acid deficiency causes delay in liver regeneration and lower levels of FGF are produced	[44]
		There is an Increase in mortality along with delay in liver regeneration in FXR-knockout mice	[45]
	D. Insulin	Insulin is necessary for EGF and HGF to work which is continuously delivered to the liver from islet -cells	[38, 46]
		Insulin's impact in reversing atrophy caused by a portacaval shunt in dogs is most likely due to the mitogenic receptors EGFR and MET	[47]
3	Factors from complex mitogenic pathways		
	A.WNT-β–Catenin	Within five minutes of partial hepatectomy, β-catenin is found in the nuclei of hepatocyte in rats	[48]
		EGFR and MET phosphorylate β-catenin at various places resulting in the protection from oxidation	[49]
		Hepatocyte proliferation is aided by β-catenin and slows but does not completely eradicate liver regeneration	[50, 51]
		Mice lacking the R-spondin receptors LGR4, LGR5 and LRP5 and LRP6 exhibit delayed liver regeneration	[52]
	B. Hedgehog	Hepatocytes express the signalling protein smoothened and the Hedgehog receptor patched 1. When the Hedgehog pathway inhibitor cyclopamine is administered to mice, liver regeneration is postponed	[53]
		Inhibiting the Hedgehog pathway in HSCs decreased Yap expression in hepatocytes and slowed liver regeneration	[54]
		transcription factor like the GPC3, the GLI1 and CD81 releases the Hedgehog ligands ends the pathway	[55]
	C. Hippo	YAP protein is controlled by Hippo pathway, through interactions with TEAD nuclear proteins suppresses the Hippo pathway	[56]
		YAP control has been linked to carcinogenesis, liver regeneration, and development	[57, 58]
		YAP interacts with TGF-1 signalling, allowing a variety of gene expression alterations linked to cell proliferation	[59]
	D. TGF-β	TGF-1 is generated by macrophages and hepatocyte-surface decorin and inhibits hepatocyte's ability to divide	[60]
		An increase in TGF synthesis, has no effect on hepatocyte proliferation during liver regeneration	[56]
		Noradrenaline in the blood is likely to downregulate the action of TGF-1 in the initial two days of regeneration	[63]
		TGF-1 influences hepatocyte gene expression during liver regeneration	[58]
		If 2-spectrin is lost, a TGF signalling pathway is linked to slowed and leads to delay in regeneration	[61]
		TGF-1 regulates the production of ECM and helps developing endothelial cells create capillaries	[62]

*HGF* Hepatocyte growth factor, *ECM* Extracellular matrix, *HB-EGF* heparin-binding EGF-like growth factor, *LSEC's* liver sinusoidal endothelial cells, *HSCs* Hepatic stelate cells, *TGFα* transforming growth factor-α, *EGFR* epidermal growth factor receptor, *CAR* constitutive androstane receptor, *FGFR4* Fibroblast growth factor receptor *CYP7A1* Cytochrome 7A1, *FGF15* Fibroblast growth factor, *TNFR* tumour necrosis factor receptor, *NF-B* nuclear factor-*κB*, *IL-6* Interlukin 6, *FEF* fibroblast growth factor, *LGR* Leucine-rich repeat-containing G-protein coupled receptor, *GPC* Grain Protein Content, *GLI* Glioma-associated oncogene homologue 1, *TEAD* Transcriptional enhanced associate domain, *YAP* yes-associated protein

## Liver model fabrication techniques

A major rising concern is towards the death of millions annually which are due to the different kinds of liver diseases. One of the reasons for patients dying is the long waiting list due to the lack of availability of donor organs. Scientists are exploring alternate solutions to overcome the rising demand; one such alternative is liver tissue engineering (LTE) principles [63]. The prime aim of liver tissue engineering is to mimic the natural liver architecture and its physiology [64]. Latest trends of liver tissue engineering are the fabrication of micro tissue structures by particularly organizing the liver cells via micro fabrication techniques and nanotechnology [65]. There are two primary uses for LTE; first, bioengineered livers may be utilised as *in vitro* models for evaluating xenobiotics (such as medications and infections) Barranger et al. developed a 3D model of HepaRG human liver cells using the CometChip technology for investigating the toxicology study as well as a disease model for liver induced toxicity [66]. Vernetti et al. created self-assembly liver model for investigating the physiology, drug safety, and disease models [67]. Second, LTE seeks to create substitutes for donor organs for *in vivo* transplantations, although being currently distant from practical use. A further advancement in the field of scaffold fabrication for tissue engineering is use of decellularized matrix derived from native organs of cadaveric donors. Uygun et al. first generated a recellularized liver graft which is transplantable in the rats from a decellularized liver biomatrix. The transplanted graft had successful liver-like functions and more efficient when comparable to normal liver *in vitro* [68].

The main ingredient of tissue engineering is the biocompatible constructs which have features like biochemical, biomechanical, and physicochemical eventually helping in providing the microenvironment for growth of cells and their differentiation which can be applied *in vivo* for liver regeneration as support devices [13, 69, 70]. These features largely influence the duration of cell survival *in vivo*, interaction of the constructs with the host post implantation, the toxic and immune responses etc. Hepatic cells are essential and there are already several potential cell sources, which are discussed later in this review paper. Other basic components of liver tissue engineering include various cell sources and supportive materials [64, 71]. The development of such constructs requires a vast knowledge spectrum covering the in-depth anatomy and functioning of organ, tissue engineering aspects, biological sciences, and use of biomaterials. Following common strategies of liver tissue engineering, researchers have started to work on the various scaffold systems, a few being—hydrogels, cell sheets, organ on chip (OOC), scaffolds etc. Many natural biomaterials (gelatin, collagen, fibrin, alginate, cellulose, heparin etc.) or synthetic polymers (polyvinyl alcohol, poly-L-lactic acids, poly-N-isopropylacrylamide, polycaprolactone etc.) are used in order to fabricate the constructs and based on the application of the construct different paracrine factors, cellular components and growth factors are included in the constructs [72–75]. Latest advancement in LTE has been exploiting the use of decellularized extracellular matrix derived from liver as they pose several advantages [76–79]. One, they have a range of biochemical constituents that can mimic the native liver tissue [77, 78]. Two, they maintain a variety of growth factors in addition to the bulk of structural proteins and polysaccharides, which may be sufficient to support cellular survival, proliferation, and differentiation with the benefit of obviating the need for extra supplementation [80, 81]. Three, they can maintain the natural liver tissue's structure, which *in vivo* provides the essential mechanical support. Fourthly, they are pro-inflammatory in nature and have minimal antigenicity, which prevents post-implantation graft rejection [82]. Numerous reviews from the past have covered the various techniques and biomaterials utilised to create tissue engineered constructions and a small attempt has been made in this paper to summarise them. [17, 83, 84]. The liver has a limited supply of cellular components, and its intricate microarchitecture and functions provide a variety of challenges [84]. Various techniques are available in the tissue engineering for fabricating and some studies for the same are summarized in Table 3.

**Table 3:** Different fabrication techniques in liver tissue engineering

Sl. no.	Fabrication style	Biomaterial used	Cell lines used	Research output	References
1	Encapsulation	Alginate-PLL-alginate	ImHH	This study developed a humanized ectopic artificial liver via tissue engineering using ImHH cells, and was able to perform stabilized functions of primary hepatocytes	[84]
2	Encapsulation	PEGDA	HH, J2–3T3mouse fibroblast, and LEC	This study generated transplantable liver grafts which supported survival of hepatocytes and optimum function with minimal ischemic damage	[85]
3	Sandwich culture	RL-dECM	RH	This study generated a functional liver from human iPSCs via transplantation of liver buds which was created *in vitro*	[86]
4	Encapsulation	RL-dECM	HFH and HH	This study encapsulated the fetal hepatocytes along with rat liver decellularized extra cellular matrix and established a healthy liver model	[87]
5	Self-assembly	–	human iHep, MSC and HUVEC	This study established a model using rat hepatocyte microbead and was found safe and improved damage significantly when transplanted intraperitoneal	[88]
6	Encapsulation	Alginate	RH	This study fabricated biomaterial platforms using liver extracellular matrix with multiple applications for liver tissue engineering in promoting hepatocytes survival and maturation. This study also demonstrated the feasibility of decellularized matrix for both 2D coating and 3D hydrogel	[89]
7	Encapsulation	RLD-dECM	RH	This study established a model using the hepatocytes and extracellular matrix from rat via encapsulation and provides a promising model for liver *in vitro* studies	[90]
8	Encapsulation	MMP-degradable PEGDA	RH, J2−3T3 fibroblasts, and LEC	This study is based on PEGDAAm-based and rat hepatocyte hydrogels for multiple applications for liver tissue engineering and model for regenerative medicine	[91]
9	Encapsulation	Alginate	HH/human iHep coaggregated with SC	This study provides a simple yet robust approach for improvement in engraftment of iHep, and is applicable to many therapies for stem cell-based studies	[92]
10	3D bioprinting	PLD-dECM	MS1 cells	This study proposes vascularized bioengineered liverswhich are size relevant, clinically transplantable *in vitro* for end-stage liver failure pateints	[93]
11	3D bioprinting	RL-dECM	porcine iHep	This study demonstrates repopulation models, and liver progeny for the role of stem-cell-derived	[94]
12	3D bioprinting	PLD-dECM	HepG2 and EA.hy926	This study established a model using the HepG2 cells and extracellular matrix from porcine which evaluated the effect of endothelialization on parenchymal cells	[95]
13	Spheroids	Alginate-collagen-PLL	AML12 hepatocyte (inner microspheres) and Rat bone marrow-derived MSC (outer sphere)	This study established the volvox spheres using the MSCs and AML12 hepatocytes which was successful in liver repair and regeneration when induced with CCl4. This study promises for effective clinical therapy for hepatic injury	[96]
14	Self-assembly	–	human iPSC, iPSCEPC, and iPSC-STM	This study successfully manufactured platform for multicellular organoid supply, which had facilitated clinical and pharmaceutical applications such as models for treatment of liver diseases	[97]
15	Electrospinning	Silk fibroin	Bone marrow/adipose tissuederived MSC	The MSCs–RSF matrices show an obvious therapeutic ability for injured liver function of mice, which is more efficient than the neat RSF scaffolds This study shows a therapeutic ability with mice liver injury on electrospinned MSCs-decellularized rat silk fibroin when compared to just decellularized rat silk fibroin	[98]
16	3D bioprinting	Collagen (endothelial cords), fibrin (hepatic microtissues)	HH, NHDF, and HUVEC	This study demonstrated 3D bioprinted engineered seeds using the hepatocytes and collagen which successfully expanded response to repair signals	[99]
17	Encapsulation	PEGDA	HH and 3T3 murine embryonic fibroblasts	This study established a 3D model using tissue engineering strategy towards and developed scalable preclinical models of liver stage for evaluating malaria infection for future applications	[100]
18	Nonwoven scaffolds	Polyglycolic acid (base material) coated with collagen	mouse/human multicellular liver organoids	This study established a liver organoid unit that composed of heterogeneous cellular population for tissue-engineered liver form *in vivo*	[101]
19	Self-assembly	–	hepatocytes, HSC, and HSEC derived from human umbilical cord blood stem cells	This study promises a new approach for *in vitro* construction of self assembled 3D human liver buds with multiple human MSC derived hepatic cell lineages. This model is useful for clinical transplantation along with regenerative medicine research	[102]
20	Self-assembly	–	human iPSC-endoderm, umbilical cordderived endothelial and mesenchymal cells	This study developed a new strategy for ALF treatment via transplantation of liver organoids that are generated from single donor-derived iPSC-endoderm	[103]
21	Scaffolds	PEGDA	iPSC-HPC	This study developed a fully defined liver organoid platform with 3D mechanical properties which can be engineered for recapitulating extracellular niche with hepatic progenitors	[104]
22	Freeze‐drying	N-isopropylacrylamide, chitosan, RL-dECM	HH or HepG2	This study promises an useful model for treatment of acute liver failure in humans using the decllularized extra cellular matrix and hepatocytes	[105]
23	Microencapsulation	collagen	RH	This study provides a 3D model for cell based-therapy along with bringing this technology closer for multiple clinical application	[106]
24	Sandwich culture	collagen	HH	This study evaluates different biochemical performance of a 3D hepatocyte culture and its application *in vivo-*like microenvironment	[107]
25	3D printing	Gelatin	HUH7 RH	This study compares disparity between gene expression in 2D culture and a 3D model and established improved expression and function of hepatocytes in 3D model	[108]
27	Freeze‐drying	chitosan and gelatin	HepaRG, LSEC and HUVEC	This study evaluated the effect of 2D and 3D cultures with different cell lines using the tissue engineering techniques and could be a potential model in different liver based *in vitro* studies	[109]
28	Freeze‐drying	Alginate/GC/heparin	Rat hepatocyte	This comparative study was to establish best condition of AL/GC/heparin sponges for hepatocytes, to provide liver-specific functions with monoculture. Cell adhesion significantly increased to GC based on AL film and can be used in future as a model	[110]
29	Freeze‐drying	Alginate and GC	MH	This study shows successful transdifferentiation rates of BMSCs to mature hepatocytes on th GC-PLGA scaffold by providing a suitable environment for differentiation. This model suggests for a possible bioscaffold for liver tissue engineering in clinical therapeutic applications	[111]
30	Freeze‐drying	Alginate, GC and heparin	MH	This study generated a 3D hepatic scaffold with mouse-induced hepatocyte-like cells and found to be a model with improved functions post transplantation *in vivo*	[112]
31	3D bioprinting	Alginate	MH	This study demonstrated a 3D model using 3D bioprinting resulting in enhanced hepatocyte functions	[113]
33	Phase separation and freezing	Galactosylated cellulose	RH	This study developed 3D model using tissue engineering application using the cellulose and rat hepatocytes for *in vitro* liver studies	[114]
34	Bioplotting	Natural ECM	iPSC	This study demonstrated a 3D culture system can increase functions of iPSC-derived hepatocytes. Further the study provides insight on scaffolds derived from ECM alone can induce further hepatocyte maturation when compared with bioplotted PLLA-collagen scaffolds	[115]
35	Pulveriziation	Natural ECM	Human hepatic stem cell	This study fabricated biomatrix scaffolds which can be used for biological and pharmaceutical studies. In future, this study promises to provide a platform for implantable, vascularized engineered tissues or organs	[116]
36	Gelation	Natural ECM	primary human hepatocyte	This study investigated the ability of 3D model with primary hepatocyte to support primary human hepatocyte specific functions *in vitro*	[117]
37	3D Bioprinting	Natural ECM	HepG2, BMMSCs	This study developed a liver decellularized extracellular matrix (dECM) bioink for 3D cell printing applications and evaluated various characteristics	[118]
38	Spheroids	PLLA	Rat hepato cyte	This study demonstrates a three-dimensional spheroids with applications for virology, toxicology, and drug development, in which metabolically active liver which proved to be more advantageous than monolayer hepatocyte cultures	[119]
39	particulate leaching	PLLA and gelatin coating	Rat hepato cyte	This study demonstrated PLLA and gelatin coating along with rat hepatocyte 3D model induced vascularization, which enabled a large attachment of cells	[120]
40	Bioplotting	PLLA and collagen	iPSC	This study used PLLA and collagen along with iPSC to establish a 3D model with clinical application using the tissue engineering *in vivo*. Further extracorporal liver assist device was established in this study	[121]
41	Rapid prototyping	PLLA	fetal liver cells	This study established a 3D structure which employs a highly accurate 3D micropositioning system using a rapid prototyping technology with hepatocytes and gelatin hydrogel having multiple applications	[122]
42	Double nozzle rapidprototyping	PCL	HUVEC	This study developed a novel 3D hybrid construct for manufacturing liver matrix using biodegradable polyurethane (PU) and a naturally derived polymer gelatin simultaneously via a double nozzle rapid prototyping technique	[123]
43	Electrospinning	PCL	human lung fibroblast HepG2	This study established a *ex vivo* model with expansion of HepG2 providing exciting possibilities for application in discovering regenerative medicine	[63]
44	3D Bioprinting	PCL-PLGA	RH hASC, RH	This study reported that a gradient of activin/TGFβ signaling on 3D bioprinted heptoblast and PCL-PLGA model	[124]
45	Double nozzle rapidprototyping	PU	–	This study established a model with endothelial differentiation of mouse ES cells induced by VEGF-C, and VEGFR3 signaling using the double nozzle rapid prototyping	[125]

*PLL* poly-L-lysine, *ImHH* immortalized human hepatocytes, *PEGDA* Polyethylene Glycol Diacrylate, *HH* human hepatocyte, *LEC* Liver enothelial cells, *RLD-dECM* rat liver derived decellularised extracellular matrix, *RH* Rat hepatocytes, *HFH* human fetal hepatocytes, *iHep* iPSC derived hepatocyte-like cells, *MSC* mesenchymal stem cells, *HUVEC* human umbilical cord-derived endothelial cell, *RH* rat hepatocytes, *MMP* matrix metallopeptidases, *SC* stromal cells, *PLD-dECM* rat liver derived decellularised extracellular matrix, *MS1 cells* murine endothelial cell line, *HepG2* liver hepatocellular carcinoma, *EA.hy926*-human umbilical vein endothelial cell line, *PLL* Poly L-Lysine, *AML12* alpha mouse liver 12 cell line, *iPSCEPC* iPSC-derived endothelial progenitor cells, *iPSC-STM* PSC-derived transversum mesenchyme, *NHDF* normal human dermal fibroblasts, *HSEC* Hepatic Sunosidal Endothelial cells, *iPSC-HPC* iPSC-derived hepatic progenitor cells, *HUH7* Hepatocyte derived carcinoma cell lines, *HepaRG* hepatic stem cell line, *GC* galactosylated chitosan, *MH* Mouse hepatocyte, *ECM* extra-cellular matrix, *iPSC* induced pluripotent stem cells, *BMMSCs* bone marrow mescenchymal stem cells, *PLLA* Poly-L (lactic acid), *PCL* polycaprolactone, *PLGA* poly(lactic-co-glycolic acid), *hASC* hepatic stellate cells, *PU* Poly Urethan

### Electrospinning

Electrospinning helps in fibre fabrication of size range from 100 nm to 0.1 µm using high voltage [126]. The high voltage is being applied on the polymeric solution and based on charge it causes repulsion (opposing the surface tension of the solution) and overcomes the surface tension that leads to formation of the jet. There is a repel created between the ejected solution and the melt, the solvent gets evaporated and the formation of fibres when the jet travels to the collector. Taymour et al. [127] generated an artificial 3D invitro model using alginate and methylcellulose.

### Phase separation

This process can be achieved at another temperature or via non-solvent to fabricate foams and membranes which are porous in nature [128]. To achieve heterogeneous scaffold structures with pores, the phase separation through nonsolvent is used [129]. These scaffolds are usually heat sensitive and not suitable for using at high temperature [130]. Further the thermal phase separation can be subdivided into solid–liquid phase separation and liquid–liquid phase separation [131, 132].

### Freeze-drying

Freeze drying is a popular method to directly convert any solvents into liable materials like solids with stability. A wide application of products derived from these methods are being used in food sciences for packaging and distributing purposes [119, 133, 134]. This process involves three major steps that are—freezing the samples at lower temperature (~ 80 °C), lowering of pressure via vacuum and finally removal of unfrozen water molecules through desorption. The scaffolds derived using this method have been widely used in the field of tissue engineering. Skardal et al. [135] created an *in vitro* liver construct with PEG based crosslinkers.

### Three dimensional (3D) bioprinting

This involved the use of biomaterials which is used to print a design 3D based on the computer aided designing. This method is widely used to obtain 3D functional scaffolds with cells for *in vivo* and *in vitro* studies [136]. With the evolving technology, the 3D bioprinting of the liver has many applications and one such is the use of 3D bioprinted material in liver transplantation [137]. Our group had recently published the detailed review on advancements in 3D bioprinting for biomedical applications [9]. Wang et al. [115] developed a 3D scaffold for demonstrating hepatocyte like cells derived from iPSC mature on bioprinted matrix.

### Self-assembly

As the name suggests, it is organizing the provided materials into design or structures via technology. It has been widely applied in fabricating various nanofibers [138, 139]. Self-assembly is a process in which nanoscale particles or materials spontaneously organise specified components into ordered superstructures that may be used in a variety of applications. Nanostructures can be created in a stirred medium by static or dynamic self-assembly. Peak et al. created self-assembly liver model for investigating the physiology, drug safety, and disease models [140].

## Bioprinting techniques used for hepatic engineering

**Fig. 5.**
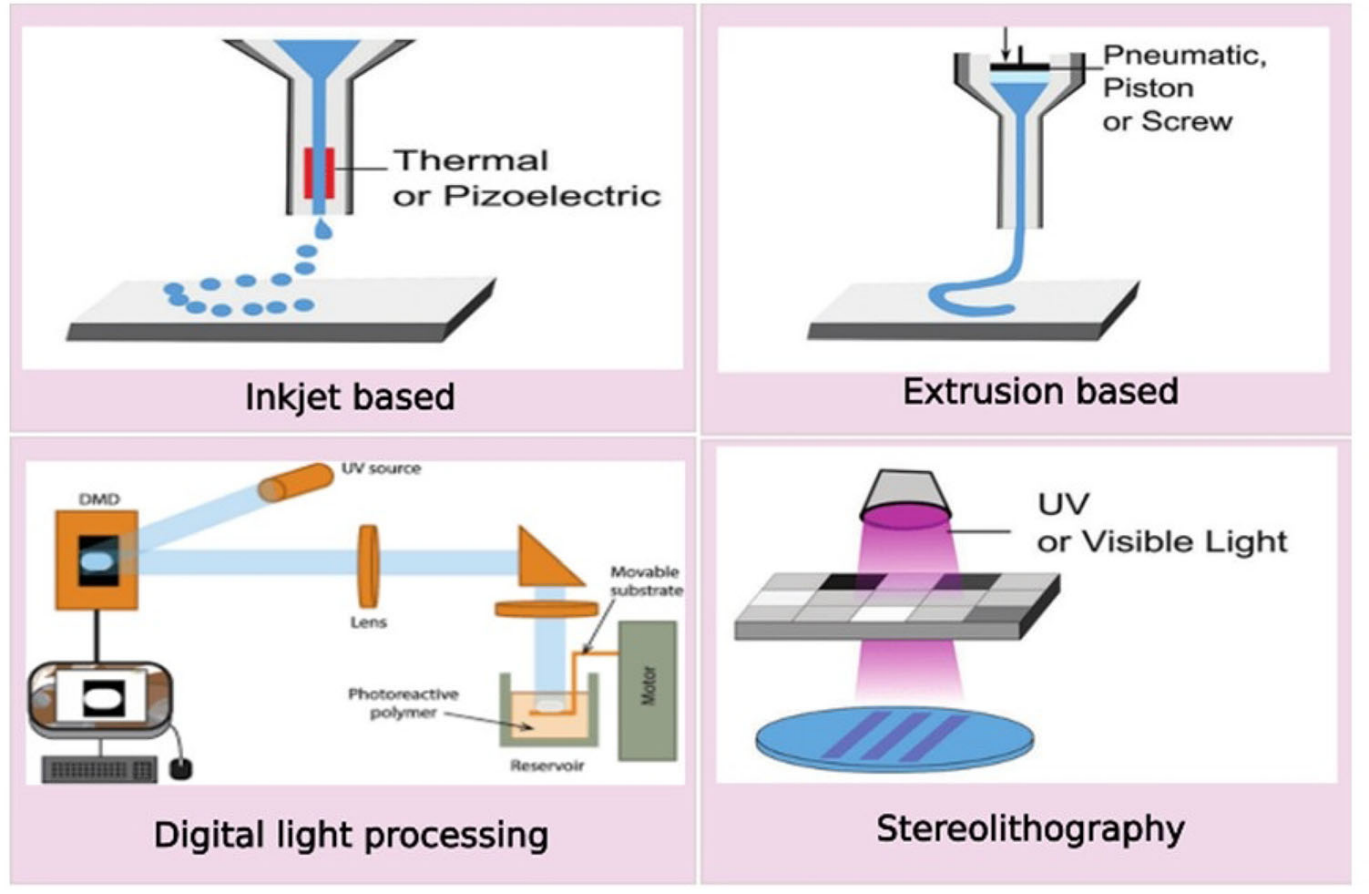
3D bioprinting techniques for hepatic liver engineering

There are various bioprinting techniques that are used to develop 3D bioprinted structures (Fig. 5). The basic components of a 3D printing system contain a filament, extruder motor, hot end, heated bed, and computer logic [141]. The various techniques are classified as extrusion based, inkjet and photocuring based techniques.

### Extrusion- based bioprinting

This technique is one of the most used 3D bioprinting techniques, it generates continuous filaments and forms 3D structures of various size, shape, and resolutions. To print scaffolds with multiple cells and vascular structures, multimaterial and coaxial extrusion bioprinting is used [142]. The parameters like temperature, pressure, velocity, cell density must be optimised to obtain good cell viability, shape fidelity and enhanced cell functionality [143, 144]. Lewis et al., precisely 3D printed gelatin scaffolds having control pore geometry. A pneumatic extrusion-based printer, EnvisionTEC (GMBH) 3D-Bioplotter was used to print the gelatin solution. The parameters such as nozzle diameter, layer slicing, strut spacing were kept constant at 200 µm, 156 µm and 700 µm respectively. The 3D printed scaffolds has 6 layers with dimensions of 15 mm*15 mm and cultured with HUH 7 human hepatocellular carcinoma cell line [108]. Kang et al., created a technique for simultaneously fabricating heterogenous, multicellular, and multi material structures. A precursor cartilage was designed and fabricated that was added to various segments of the printer. The different types of bioinks were added to the cartridge and then placed into the syringe. ECs and HepG2 cells were used to fabricate the co-culture model. The use of heterogeneous cells for developing hepatic lobule increased the cell functionality in comparison to homogenous cell printing [145]. Billiet et al., fabricated 3D printed microporous scaffolds using gelatin methacrylamide using a bioplotter dispensing system to develop the scaffolds. The printer has three axes for pneumatically dispensing the deposit cell laden hydrogel on the platform. To support the temperature of the gelatin, homogenous plotting temperature was adjusted till the dispensing needle and the stationary platform was equipped with cooling elements such as Peltier element and HepG2 cells with seeding density of 1.5*10^6^ cells/ml were seeded. Plotting speed, air pressure and temperature were optimised and a scaffold of dimensions, 1–3 mm thick with 13*13 mm dimensions were fabricated [146].

### Inkjet-based bioprinting

The basic principle of inkjet based bioprinting is the formation of individual droplets to form the construct [147]. There are three main approaches to droplet formation such as thermal, piezoelectric and electrohydrodynamic methods. This method is better than extrusion based in terms of controlling the biological elements but the limitation of this technique is the use of only low viscosity materials [148]. Lee et al., fabricated 3D hepatic block scaffolds using neutralised type 1 atelo collagen solution was bioprinted and it showed differentiated human adipose stem cells to hepatocyte-like cells. The scaffolds were designed in a 3D bioprinter with 29G blunt nozzle and fixed with a moving speed and pressure of 2 cm s^−1^ and 150 kPa respectively [149]. Parsa et al., improved the reliability of the droplet formation that is inconsistent in inkjet-based 3D printing by stirring and gentle agitation. The researchers worked with type I rat-tail collagen and fabricated a hydrogel and seeded with HepG2 cells. The inkjet printer was piezoelectrically driven and had a nozzle of size 100 µm. The reservoir of the printer was loaded with HepG2 and seeded in concentration of 200,000 droplets or 10,000 cells on the gelled hydrogel [150]. Moya et al., developed an oxygen monitoring system on the liver organ on a chip (OOC) by placing multiple sensors on the thin membrane inside the liver OOC. The sensors were printed using an inkjet printer along with the microfluidic channel. Three kinds of ink formulations were used to print the DO sensors- low-curing gold, silver, and dielectric photoresist SU-8. For the printing process, a primer layer was formed to seal the porosity of the membrane where the sensors will be placed. The steps of printing the DO sensors were first, the printing of SU-8 ink as the primer layer, then printing of two SU-8 layers with spacing between the drops (DS), gold elements and counter elements were printed next also with DS of 15 µm. Silver elements were printed next to develop the electrode structures that form pseudo-reference electrodes with DS 30 µm. Primary human and rat hepatocytes were used to prove the concept and oxygen consumption rate was calculated using carbonyl-cyanide-4-(trifluoromethoxy)phenylhydrazone. The study demonstrated that the inkjet printing technique is a feasible technique that can be used for the integration of the sensors for evaluating the metabolic activity of cells [151].

### Photocuring-based bioprinting

In this technique, the photosensitive hydrogel is cured using light irradiation and creates complex structures in comparison to extrusion and inkjet-based techniques [152]. There are two types of photocuring techniques-stereolithography and digital light processing. Yu et al., used specific decellularized bioink to fabricate patient specific tissues using digital light processing based bioprinter. The bioprinter was an in-house developed DLP based system that had five main components namely-UV light source, computer for sliced image- flow, a digital chip that helps in modulating the UV light and is comprising around 2 million micro-mirrors, projection optics set and a linear stage to position the sample and control the projected image and focal plane. The dimensions for the liver construct were 3 × 3 × 250 µm with a lobular pattern and small regions showcasing central vein and portal triad. hiPSC-hepatocytes were added to the dECM bioink in concentration of 30 million cells/ml and the construct was printed on a 24 well plate which was then maintained further according to the requirement of the cells in the culture. This approach allowed rapid bioprinting with accuracy and precision with maintenance of relevant functional properties [153]. Grix et al., provided with evidence for bioprinting a liver organoid. They utilised the stereolithographic printing approach and used HepaRG and human stellate cells and the liver construct was made up of two materials- PEG for the channels and GelMA for holding HepaRGs and SteCs of 4 mm thickness. Using blue illumination, the layer printed was photopolymerized directly. In one printing process, six tissue models were printed parallelly with each model containing 10^6^ cells [154]. Ma et al. fabricated 3D hydrogel that were having hiPSC-HPCs using stereolithographic based bioprinting system. A customised bioprinting system was used with a DMD system, LED light source and movable stage. The triculture 3D model was printed with first layer of hepatic cells then another layer of complementary cells. The hepatic model was able to provide *in vitro* maturation of hepatic cells in the microenvironment of hepatic lobule microarchitecture [155].

## Bioinks for hepatic engineering

**Fig. 6:**
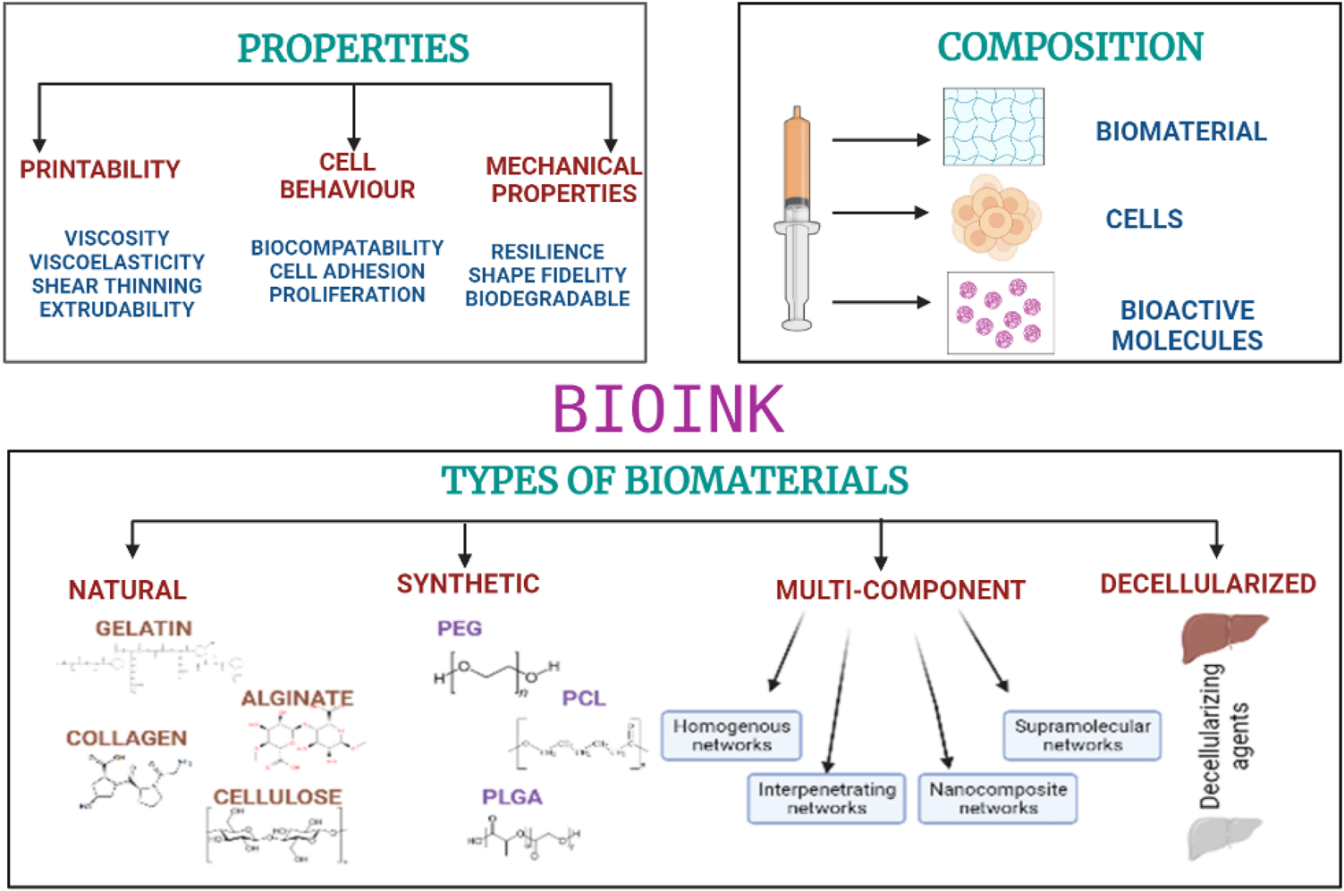
Bioinks-properties, composition and types

Bioinks are the major component of 3D bioprinting and comprises cells, biomaterial, and other biological factors. The materials must be biocompatible, biodegradable, non-toxic, non-immunogenic. Based on the requirement of the construct, the cells and materials are selected, for example, models for drug designing, regeneration, disease modelling, etc. The cells should interact with biomaterials and be able to retain their biological functions. Figure 6 illustrates the types of bioink along with its composition and properties. Hepatocytes are the most abundantly present parenchymal cells in the liver and along with it there are Kupffer cells, endothelial cells, and hepatic stellate cells. For constructing liver tissue models, cell lines are available which utilises the primary human liver cells.

### Natural polymers based bioink

The naturally occurring polymers that are the preferred choice for printing liver tissue owing to their properties such as biocompatibility, biodegradable, non-toxic, support angiogenesis and organogenesis. Gelatin, which has a single chain of polymers that is obtained by partial hydrolysis of collagen is the most sought after natural based polymer for tissue engineering applications. Rapid solubility in biological buffers, adaptability for crosslink, thermosensitivity, cell recognition property, and non-toxin makes gelatin the highly researched compound for 3D bioprinting [154–158]. Roopesh et al., developed a high throughput method that helps in fabrication of liver parenchymal microtissues (LPMT) with use of GelMA and HepG2 cells. The LPMT was fabricated by the technique of hanging drop culture chamber, a novel technique that was developed by the team. HepG2 droplets were suspended on polyethylene terephthalate substrate and this substrate was then kept inside the chamber where LPMTs were obtained. GelMA was used to provide stability to microtissue by sandwiching the LPMT between GelMA hydrogel enhancing the liver functions and response to insulin stimulation. The GelMA sandwich of LPMTs showed better activity of urea synthesis, albumin secretion, P450 cytochrome activity in comparison to only LPMTs in suspension [159]. Alginate, obtained from seaweed algae, and is a negatively charged polysaccharide having made up of two copolymers- guluronic acid and mannuronic acid, that helps in providing the gelation and the flexibility to the material [160]. Crosslinking of alginate is only possible via divalent cations, as physical gelation cannot be supported due the sol–gel temperature being below 0°C [161]. Alginate is a low-cost material with sufficient biocompatibility to help in development of *in vitro* scaffolds. Xie et al., developed a novel modelling system technique for generating hepatorganoids using HepaRG cells. Patient samples of HCC were obtained and primary HCC cells were isolated. Gelatin and sodium alginate were used as materials to which cells were added. The bioink was added to the 3D cell printer and layer by layer construct was printed and *in vitro* analysis was performed. The 3D printed- HCC model showed uniformity in cell density and distribution, the biological characteristics were retained, and this model can be used to screen drugs for various HCC patients [162]. Collagen, is one of the most abundant proteins, is highly biocompatible and due to presence of RGD motifs support the cell attachment as well [163]. Collagen is considered for encapsulating hepatocytes. Crosslinking can be done via chemical crosslinkers, changing pH and temperature [164]. The mechanical strength of collagen is very poor, to overcome this, it is added with supporting materials such as polycaprolactone (PCL). Mazzocchi et al., developed a bioink for fabricating 3D printed liver tissue construct using collagen type I and liver cells like hepatocytes and stellate cells. Methacrylated collagen type I and thiolated hyaluronic acid was used to prepare printable bioink and Allevi 2 bioprinter was used for liver model printing. To study the functionality, primary human hepatocytes were bioprinted in a four-spoke structure and cell viability and functional tests were performed by adding acetaminophen. The liver construct was able to maintain urea and albumin production and responded well to APAP [165]. Cellulose is a non-biodegradable and biocompatible natural polymer that does not support cell adhesion [166] and is being used as bioink in two forms, carboxymethylcellulose and nanocellulose [167]. The former properties can be tuned depending on the degree of methylation [168]. Nanocellulose is biocompatible and successfully used to 3D print structures. It is either present in the state of crystals or fibres in a nano-structured form [169]. Wu et al., fabricated a bioink consisting of alginate, cellulose nanocrystal and GelMA to construct liver tissue. A honeycomb scaffold was 3D printed using micro-extrusion based printing and the matrix between the scaffolds were filled with GelMA hydrogels containing HepG2 cells. The construct showed intracellular interactions and alignment along with albumin secretion [170].

### Synthetic polymers based bioink

The tuneable properties have made the synthesised polymers to be used for 3D printing of liver. The mechanical and physicochemical properties of such materials are tailored and can be controlled by chemical modifications. Most commonly used synthetic polymers for 3D printing of liver are polyethylene glycol (PEG), polycaprolactone (PCL) and poly (lactic- co- glycolic acid) (PLGA) are all FDA approved polymers known for biocompatibility and widely used for various tissue regeneration. Chen et al., fabricated human ectopic artificial livers (HEALS) by using PEG and human hepatocytes and implanted in nude mice. Drug metabolising enzyme functions were tested, HEALS were able to express them and also showed potential in screening of compounds such as Cytochrome P450. This kind of model applications include providing a supportive microenvironment, delivery vehicle and also a barrier for rejection-delaying process [85]. Seng Ng et al., developed a 3D printed hexagonal model using polyethylene glycol (PEG) and human foetal liver cells that was inspired by natural liver architecture to assess antiviral agents, understand specific drug metabolism and also detect specific drug hepatotoxicity. The model showed differentiation of specific factors and also maintained the advanced functions [171]. PCL is a hydrophobic homopolymer that is synthesised by polymerization of ring structure of caprolactone. Grant et al., fabricated a 3D printed scaffold using Electrospun PCL, ECM and HepG2 cells. ECM layer was placed on PCL scaffold layer which was cut from the Electrospun fibres and then onto that was then seeded with HepG2. The construct shows potential in altering the production of ECM and also future in liver tissue engineering [172]. Park et al., fabricated an anastomosis device by the name absorbable vascular anastomosis device using PLGA and consisted of two inner rings and a coupler. The device was able to maintain its shape for 3 weeks, and the device was implanted in mini-pigs and after 4 months autopsy showed that the device was completely absorbed [173].

### Multicomponent bioink

As the name suggests, multicomponent bioink comprises two or more biomaterials having good rheological properties that can be adjusted during and after printing. There are four types of multicomponent bioinks which includes homogenous networks that forms covalent crosslinking with each other, interpenetrating networks forms independent crosslink networks and gets intertwisted with each other, nanocomposite networks has increased stiffness by adding nanocomposites and the supramolecular networks have reversible functional groups [158, 174–177]. Taymour et al. fabricated a 3D bioprinting based concept to develop a 3D core–shell liver model. Alginate and methylcellulose were used as biomaterials to form this multicomponent bioink along with HepG2 cells and fibroblasts where they acted as supportive cells. There was proper cell attachment, viability, proliferation of the cells in the core shell scaffold [178]. Hiller et al., formulated a bioink that had alginate, gelatin and human extracellular matrix along with HepaRG liver cells and used Pneumatic extrusion printer to fabricate the 3D model. The construct was stable, viable and had desirable properties of metabolic functions of the cells and along with it, the model also showcased the efficient adenoviral replication [179]. Leva et al., used direct laser induced forward transfer technique to develop a 3D printed scaffold for liver regeneration. Collagen-GAG scaffolds were prepared using Huh-7 cells and then analysed quantitatively and qualitatively. The cells were viable for 2 and 24 h in the desired pattern and were properly adhered to the scaffold [180]. Khati et al., used gelatin and polyethylene glycol to develop a 3D printed model for liver. Decellularized liver matrix bioink containing HepG2 cells. To enhance the stability and application, the bioink was cross linked chemically via mushroom tyrosinase and due to this, an increase of 16-fold was observed in the viscosity of the bioink and storage modulus in comparison to bioink without crosslinking. The grid model was able to support cell proliferation and cell adherence [181].

### Decellularized extra cellular matrix based bioinks

The decellularization process removes cellular content on the tissues and organs and preserves the integrity of the native extracellular matrix and its components [182]. There are few criteria that should be fulfilled for a tissue to be considered as fully decellularized firstly, DNA content less than 50 ng/mg, 200 bp or smaller DNA fragment length, second absence of visible cellular content in histological characterizations like H&E staining and DAPI [183, 184]. The physical method of decellularization in which physical parameters are targeted such as temperature, pressure and pH to burst the cells. Some of the techniques for physical methods are freeze–thaw, mechanical agitation, supercritical CO_2_, high hydrostatic pressure [185, 186]. The chemical method involves use of chemical agents such as detergents, ionic liquids, acids, bases to decellularized the tissues by disrupting protein- protein interactions, disrupting the nucleic acid compositions and solubilising cell membranes [178, 186, 187]. Enzymatic method is another way of decellularizing in which enzymes like proteases, nucleases, chelating agents are used which cleaves the cell matrix adhesions and cleaves proteins and peptidases [178, 186]. To evaluate successful decellularization of any tissue, it is important for qualitative assessments like H&E staining, DAPI and quantitate DNA, sGAGs, collagen, elastin, or other ECM.

Many studies have used dECM bioink to 3D bioprint liver constructs. Skardal et al., developed a 3D bioprinted liver spheroid using hyaluronic acid, gelatin and decellularized extracellular matrix. The dECM tissue was prepared using Triton X-100 and NH_4_OH and then lyophilised for further use. Hepatocytes, stellate cells and Kupffer cells were combined in a ratio of 80:10:10 and spheroids were formed on 96 well plates. PEGDA and PEG were used as crosslinkers. Multiple crosslinking steps were done including thiol-acrylate and UV light. The 3D liver construct developed showing functional albumin and urea output [187]. Lee et al., fabricated bioink for liver 3D printing applications using decellularized extracellular matrix employing Triton x-100, NaCl, and SDS. Porcine derived collagen was used along with dECM with HepG2 and BMMSCs. Cell behaviour was analysed by cell viability assay. Enhanced liver functions were observed on constructs fabricated using HepG2, dECM and collagen [71]. Mao et al., fabricated a liver microtissue using liver specific bioink having GelMA, dECM and hiHep cells. Tissue was decellularized using triton X-100, 2% SDS and ultrapure water. For solubilising, acetic acid and pepsin was used which completely dissolved the lyophilised decellularized tissue powder and then mixed with GelMA. To the dECM/GelMA ink hiHep cells were added in concentration of 2.5–3.0*10^6^ cells/ml and cell laden bioink was deposited as a small droplet using a DLP based bioprinting system. The cells expressed hepatic function in the 3D bioprinted liver microtissue [188].

### Cell sources for bioinks

Liver tissue engineering has multiple applications, but the main target is to achieve the liver microenvironment on the material being fabricated. Non-parenchymal cells are now thought to be just as crucial for the success of liver tissue engineering as the typical hepatocyte cells. The major downside of using these cell lines is that the isolation and their maintenance *in vitro* have low yields which also limits their application [189]. Researchers have been attempting to eliminate different liver cells by using stem cells to replace it with other cell types in an effort to overcome this obstacle. Human Umbilical Vein Endothelial Cells (HUVEC) are produced from adult stem cells, induced Pluripotent Stem Cells (iPSCs), and embryonic stem cells are used in a number of research [188, 190]. HSCs have been derived from iPSCs [191], cholangiocytes, the primary building block of the biliary network, are produced in the liver from foetal or progenitor cells [192, 193]. The cell lines are an important source as it contributes majorly in engineering of the liver tissue [194]. In spite of all the findings and advancement in technology, there are multiple complications that arise in the protocol for the differentiation and efficiency of the liver cells that are derived from the stem cells. Another major concern rises as there are ethical concerns and the risk of carcinogenesis [1, 49, 79, 195]. Below are a few of the cell line sources which are potential sources to derive the liver cells.

Human Primary hepatocytes (hPHs) are the leading mature cell lines for the liver and possess the highest proliferation rate *in vivo* [194, 195] but they tend to lose their function *in vitro* leading to dedifferentiation. Few studies have proved that the viability and functions can be enhanced if the cells are aggregated in the plates [158]. This led to another challenge of preventing the necrosis taking place in the centre of aggregated cells and to improve the supply of oxygen and nutrition [16, 196, 197]. Another approach that researchers have figured out is to culture the HPHs with other cells like sinusoidal epithelial cells and stellate cells and fibroblasts [198, 199].

Primary hepatocytes are widely used from various animal sources that include rat and porcine. They are famous as they are readily available but their application is very limited due to compress compatibility for which specialised conditions shall be provided (encapsulation). A very good example of the encapsulation is the encapsulation of Fetal liver cell (FLC) and β-Fibroblast Growth Factor (βFGF) which showed improved functioning [200]. Primary hepatocytes from rats are used in few studies which involve bioprinted scaffolds. Another example is the use of hPHs and primary hepatocytes were encapsulated with alginate hydrogel [201]. One of the major drawbacks is that the process of isolation is very difficult and the viability of cells in tissue engineering application is less.

Stem cells (SCs) and Embryonic stem cells (ESC) are increasing interest in the use of stem cells as they have high potential for differentiation, proliferation and self-renewal [202–204]. The expression of genes that are specific to the liver were studied by Zhao et al. by the differentiating embryonic stem cells; moreover, the increased functional activities were shown by these cells when they were implanted in mice liver. There was observation of markers which are specific to the liver, absorption of lipoproteins, secretion of albumin and storage of glycogen [205]. The major drawback is that the cells have higher proliferation rate which leads to formation of tumours and hence it’s difficult to maintain them in undifferentiating state [206, 207].

Induced pluripotent stem cells are the undifferentiated pluripotent stem cells that have been reprogrammed via various reprogramming ingredients and are used because of their specificity for the patients [208–211]. These cells can be isolated from mice, humans, and porcine [212–217]. For proper functioning of these cells there is an additional requirement of adipose‐derived stem cells and human umbilical vein endothelial cells for support and structure of the liver. The major drawback is that these cells showcase different phenotypes due to the difference in the epigenetic memory of iPSCs [218].

The multipotent mesenchymal stem cell has multiple sources such as the adipose tissues, bone marrow, umbilical cord blood and tissue, amniotic fluid, Wharton jelly, dental pulp, etc. [83, 219–221], they are in the state of undifferentiation until the time the body requires them but at the same time these cells tend to lose their capacity to proliferate when they are cultured [222–225]. A study proves that the encapsulation helps these cells to have higher levels of cytokines when compared to the *in vitro* tests as there is a lack of surface receptors [226–229]. Given a chance to compare the ESCs and iPSCs, iPSCs do not have potential for proliferation [230, 231].

Foetal Liver Cells (FLC) are considered a suitable hepatocyte substitute due to the ability to form a colony unit in cell culture. Suzuki et al. showcased that these cells differentiated into cholangiocytes or hepatocytes which are the parenchymal cells of the liver [125]. Human adult liver stem cells (HLSCs) are in the small branches of bile canaliculi in a matured human liver [232]. They have great potential to differentiate into insulin producer cells, endothelial and bone cells, however they can be differentiated into hepatocytes based on the requirements [233]. Bone Marrow-derived Very Small Embryonic‐Like Stem Cells (BM-VSEL) migrate from the bone to the site of liver injury and express many hematopoietic stem cells. Studies have demonstrated that these cells have capacity to differentiate into hepatocytes. In one of the studies, they attempted to use hepatocellular transplantation from living donors to those in need for liver transplantation [234].

## Applications of 3D bioprinting for liver diseases

Liver plays a major role in the metabolic process of the body and holds important functions such as protein synthesis, bile secretion, regulates blood clotting, albumin production, regulates amino acids, resists infections and many more. Although the liver has a great capacity to regenerate naturally, an excess level of cell damage leads to irreversible damage to liver cells [235]. Severe liver impairments are observed in liver diseases and hence it is important to develop an *in vitro* liver disease model to study the diseases and understand the underlying molecular pathways associated. This also helps in performing drug screening and toxicity tests. Also, liver transplantation is an effective treatment in many end-stage liver diseases. In the current scenario, organ shortage is a global problem and tissue engineering techniques are aimed at developing artificial organs to help address this issue [236]. 3D bioprinting shows a huge potential in fabricating an artificial organ with close biological resemblance that can be used for disease modelling, drug screening and transplantation in humans in the near future.

### Pre-clinical and clinical trials

There are currently multiple active research studies that are using 3D bioprinting techniques specifically for the purpose of engineering liver tissue. These clinical trials involve the application of 3D bioprinting technology to create functional liver tissue in a controlled laboratory setting. The trials aim to develop innovative approaches to regenerate or repair damaged liver tissue, potentially leading to advancements in the treatment of liver diseases, transplantation, or drug development. These trials are conducted under rigorous scientific protocols to evaluate the safety, efficacy, and feasibility of 3D bioprinting methods for liver tissue engineering. However, it is still in the early stages of development, and it will take several years before 3D bioprinted liver tissue becomes available for clinical use.

Ernesto et al., evaluated the impact of single cell dispersion, spheroids of iPSc derived parenchymal cells and non-parenchymal cells in both systems tested for liver tissue functionality. Printed spheroid constructs showed greater cell survival and hepatic functions in comparison to single cell dispersion. Their study demonstrated the advantages of using spheroid based bioprinting which can be further used for developing accurate disease models [237]. Two researchers from Wake Forest Institute of Regenerative Medicine have successfully bioprinted a vascularized liver tissue with greater than 85% cell survival rate for 30 days [237]. These two teams have won the vascular tissue challenge by NASA. The implications of their study have applications in disease modelling, drug testing and probable transplantation in the future [238].

A few pre-clinical studies on 3D bioprinting liver are mentioned in Table 4 and clinical trials are listed in Table 5.

**Table 4:** Pre-clinical trials on 3D bioprinting liver

Sl. no.	In-vivo/in-vitro model	Bioprinter	Biomaterial	Cells	Growth factors	Study period	Application	References
1	Mice	Extrusion based	Sodium alginate	HepaRG cells	–	7 days	Liver Transplantation	[239]
2	Grid	Extrusion based	Sodium alginate	HepG2 cells	–	10 days	Disease modelling	[240]
3	Grid	Extrusion based	Alginate cellulose nanocrystal	Hepatoma cells, fibroblasts	–	3 days	Bioink	[241]
4	Mice	Extrusion based	Collagen-chitosan	HL-7702 cells	Hepatocyte growth factor (HGF)	14 days	Reconstruction of tissue	[143]
5	Grid	Extrusion based	135 ACG (Alginate, cellulose nanocrystal, GelMA)	NIH-3T3 cells, HepG2 cells	–	14 days	Bioink	[155]
6	Grid	Extrusion based	Porcine derived dECM, gelatin, PEG	HepG2 cells	–	7 days	Bioink	[137]
7	Wheel with spokes	Extrusion based	Hyaluronic acid, collagen-I	primary human hepatocytes and liver stellate cells	–	7 days	Bioink, drug testing	[242]
8	Hexagon	Light based	GelMA, porcine derived dECM	HepG2 cells	–	7 days	Disease modelling	[243]
9	Tri lobular structure	Extrusion based	PVA, dECM	NIH-3T3 cells, HepG2 cells	–	7 days	Bioink	[244]
10	Grid	Inkjet based	Galactosylated alginate	Hepatocytes	–	Unknown	Bioink	[245]
11	Liver like model	Extrusion based	Liver extracellular matrix	Human adipose mesenchymal stem cell derived HLCs, HUVECs, HHSCs	–	7 days	Drug testing	[246]

*HepaRG* hepatic cell line, *HepG2* human hepatic carcinoma cell line, *HL-7702* human liver cancer cell line, *NIH-3T3* Embryonic mouse fibroblast cell line, *HLCs* hepatocyte like cells, *HUVECs* Human umbilical vein endothelial cells, *HHSCs* Human hepatic stellate cells

**Table 5:** Clinical trials on 3D printing liver

Sl. no.	Clinical trial study title	Country	Study start date	Status	Registered at
1	Applicability of 3D printing and 3D digital image reconstruction in the planning of complex liver surgery (LIV3DPRINT)	Spain	2017 June	Unknown	Clinicaltrials.gov
2	Application of 3D visualization and 3D printing in the hepatobiliary and pancreatic surgery	China	2017 March	Unknown	Clinicaltrials.gov
3	Application of 3D printing in laproscopic surgery of liver tumors	Poland	2017 April	Active	Clinicaltrials.gov
4	Value of 3D printing for comprehension of Liver surgical anatomy	China	2017 July	Completed	Clinicaltrials.gov
5	Clinical efficacy of stereotactic radiotherapy and microwave ablation for liver metastases from colorectal cancer	China	2019 June	Unknown	Clinicaltrials.gov
6	Patient-Individualized resection planning in liver surgery using 3D print and virtual reality (i-LiVR)—a study protocol for a prospective randomized controlled trial	Germany	2022 January	Active	German clinical trials
7	Individualised Liver resection planning using 3D printing and virtual reality	Germany	2022 May	Active	German clinical trials

### Transplantation

3D printing is a useful tool for anatomical visualisation for surgical planning. It is particularly useful in case of complex structures and surgeries. Zein et al. printed liver models with vascular network and biliary structures mimicking the native livers of six patients—three living donors and three recipients (patients with cirrhosis) for living donor liver transplantation. TangoPlus/VeroClear synthetic material was used for printing. The objective was to generate accurate models to help minimise operative complications by facilitating pre-operative planning to identify vascular networks and biliary anatomy in the patient’s models [247]. Yukio et al., developed a 3D printed liver model for hepatectomy which improved the visibility during the procedure, helped simulate the resection line [248]. Studies showed that these 3D printed liver models have proven to be useful for pre-operative planning [249–255]. So far, clinicians have used 3D printed models for pre-planning surgeries before transplantation. The development of a 3D bioprinted liver is not at an advanced functional stage that it can be used for human liver transplantation.

The clinical translation of 3D bioprinted liver constructs for transplantation faces several challenges such as biocompatibility and immunogenicity, vascularization, maintaining structural integrity and mechanical strength, long term functionality and integration, scale up manufacturing, regulatory approval and standardization. Ensuring that the 3D bioprinted constructs are biocompatible with the recipient's body and do not trigger an immune response is crucial for successful transplantation. The materials and cells used in the bioprinting process must be carefully selected and engineered to minimize immune reactions and promote integration with the host tissue. Adequate vascularization is essential for the survival and functionality of large, complex 3D bioprinted constructs. Creating a network of blood vessels that can efficiently deliver nutrients and oxygen throughout the construct remains a significant challenge. Without proper vascularization, the transplanted tissue may suffer from insufficient nutrient supply and impaired functionality. 3D bioprinted constructs need to exhibit sufficient structural integrity and mechanical strength to withstand transplantation procedures and function in the recipient's body. Ensuring that the constructs maintain their shape, integrity, and mechanical properties over time is critical for their long-term success. Scaling up the production of 3D bioprinted constructs to meet the demands of clinical transplantation is a challenge. Manufacturing processes need to be optimized to ensure reproducibility, consistency, and cost-effectiveness while maintaining the quality and functionality of the constructs. Meeting the rigorous regulatory requirements for clinical use of 3D bioprinted constructs is a complex and time-consuming process. Establishing standardized protocols, safety assessments, and quality control measures is necessary to obtain regulatory approval for clinical trials and eventual commercialization. Ensuring the long-term functionality and integration of the 3D bioprinted constructs with the recipient's native tissue remains a challenge. Construct durability, cell viability, functional performance, and the ability to integrate into the host tissue are critical factors for successful transplantation outcomes. Addressing these challenges requires ongoing research, technological advancements, collaboration between different disciplines, and rigorous preclinical and clinical evaluations to establish the safety and efficacy of 3D bioprinted constructs for transplantation.

### Disease models and drug screening

Healthy liver tissue models are needed to study the molecular state of a healthy liver and how these change in a diseased liver. Hence, a healthy model is fabricated, then the disease is induced and both the models are compared to study the physiological changes. This helps in understanding the underlying mechanisms and pathways that caused the disease. Liver diseases such as cirrhosis, liver cancer, fatty liver disease, hepatitis, etc. pose a serious threat to human health globally [256]. To reduce the morbidity associated with these diseases, an accurate disease model is required to screen the drugs efficiently. Often, animal models and 2D cultures are used for this purpose but they do not mimic human physiology and greater side effects are observed when translated to humans. These methods do not represent the human metabolic microenvironment either. Also, these methods are time consuming and expensive. Accurate liver disease models can be useful in development of new drugs and screening existing drugs [257–260]. Disease models help us understand the efficiency of drugs. During the drug development process, most drugs are not successful due to the liver injury caused by them i.e., hepatotoxicity. Since, liver is an essential organ for detoxification and drug metabolism, it is important to have relevant liver models to test for drug toxicity. 3D bioprinting technology helps overcome current challenges of tissue engineering and drug development by providing a personalised tissue/organ model [261]. 3D bioprinting is preferred since it is a more precise way to develop a construct in-vitro. This also helps replace preclinical animal testing in the near future. This technology helps mimic the human liver in-vitro for the purpose of developing disease models to understand the disease progression and the cause of it, to screen and test drugs which are efficient and are not toxic [133, 262, 263].

Xie et al., established a modelling system to fabricate hepatic organoids using 3D bioprinting technology with HepaRG cells for drug screening. Gelatin and sodium alginate were used in the bioink along with patient derived hepatocellular carcinoma cells. Four targeted drugs were tested for efficacy. The established model was designed to retain the natural functions of a liver and prolong mice survival after transplantation [162]. Deborah et al., developed a bioprinted liver model with patient derived hepatocytes and non-parenchymal cells. Two drugs- Trovafloxacin and levofloxacin were tested on the bioprinted model. It was demonstrated that the bioprinted model could model drug induced liver injury and the results showed that they mimicked human drug response at the tissue level [264]. Studies in 3D bioprinting of liver are summarised in Table 6.

**Table 6:** 3D bioprinted liver models

Sl. no.	*In-vitro* model	*In-vivo* model	Bioprinting type	Biomaterial	Cells	Study period	Application	References
1	Square	–	Extrusion-based	Alginate	Mouse primary hepatocytes	3 weeks	mimic liver structure	[265]
2	Square	–	Extrusion-based	GelMA	HepG2	–	Exploring the material properties of GelMA and scaffold architecture	[140]
3	Square	Rats	Extrusion-based	Collagen solution derived from porcine skin	AHLCs differentiated from hASCs	4 weeks	Regeneration of damaged liver tissue	[129]
4	Square	–	Extrusion-based	Collagen, PCL	Primary rat hepatocytes; human endothelial cells; HLFs	2 weeks	Fabricating liver scaffold with vascular formation	[266]
5	Square	–	Extrusion-based	Liver dECM	HepG2	1 week	Developing a new biomaterial for 3D bioprinting	[113]
6	Square	–	Digital light processing	Liver dECM	hiPSC-derived cardiomyocytes and hepatocytes	1 week	Construction of hepatic lobular structure scaffold	[148]
7	Round	–	Extrusion-based	Gelatin	HUH7	14 days	Investigating the effect of the hole size and the angle between the lines on cells	[139]
8	Lobule shape	–	Digital light processing	Gelatin and PEG	HepaRG and human stellate cells	2 weeks	A hepatic lobule equivalence consisting a hollow-channel system for perfusion	[267]
9	Lobule structure and vascular structure	–	Digital light processing	GelMA and Glycidal methacrylate-hyaluronic acid	hiPSCs and HUVECs	1 week	Printing a hepatic lobular structure scaffold containing two liver-related cells	[150]
10	Square	–	Extrusion-based	Alginate and gelatin	HepaRG cells	3–4 weeks	3D-bioprinted hepatorganoids making mice have human-specific drug metabolism function	[268]
11	Round	–	Extrusion-based	Alginate, gelatin, and collagen	Hepatocyte and endothelial cells	7 days	A preset extrusion bioprinting technique to create sophisticated liver lobule models	[107]
12	Square	Mice	Extrusion-based	Alginate	Mouse-induced hepatocyte-like cells	7 days	The scaffold with miHeps transplanted into mice as a liver damage model	[176]
13	Square	–	Extrusion-based	Alginate/gelatin bioink with hECM	HepaRG	1 week	Exploring the effect of hECM on the mechanical properties and biocompatibility of the materials	[269]
14	Mini liver	–	Extrusion-based	NovoGel 2.0 Hydrogel	Hepatocytes, HSCs, and endothelial cells	4 weeks	A bioprinted liver model to model liver injury leading to fibrosis	[270]
15	Square	Mice	Extrusion-based	Alginate, gelatin, and fibrinogen	HepG2	-	To compare drug sensitivities of several anticancer drugs to the HepG2 cells in both 3D models and 2D monolayers	[187]
16	Round	–	Digital light processing	GelMA + liver dECM	hiHep	1 week	Development of liver specific bioink	[182]
17	Liver on chip	–	Extrusion based	Gelatin, collagen, PCL	HepG2, HUVEC	1 week	one-step fabrication of lover on chip with a 3D bioprinting technology	[271]

*AHLCs* hepatocyte-like cells, *hASCs* human adipose stem cells, *HLFs* human lung fibroblasts, *hiPSc* Human-induced pluripotent stem cell, *HUH7* hepatocellular carcinoma cell line

### Liver devices

3D printed devices simplify the therapeutic procedures of liver surgeries and also help in diagnosis and detection of diseases. Samar et al., developed an acoustic and electrochemical biosensor for liver cancer cells detection. The developed biosensor recognizes the highly expressed tumour marker CD133 [271, 272]. Shin-Young et al., developed a 3D printed implantable drug delivery device and was evaluated in-vivo for safety and usability. The container in the reservoir was 3D printed which contains a semi-permeable membrane for administration of drugs. The study concluded that the system was safe for acute liver failure treatment and that various drugs could be administered via this 3D printed device [273]. Organ-on chip devices have gained a lot of traction these days for in-vitro studies of pathophysiological processes for drug screening. Micropumps are used to simulate the in-vivo-like fluid flow and they help in transport of nutrients. Amar et al., designed and developed a micropump by 3D printing it for continuous perfusion of nutrients in a liver-on-chip model [274]. Gou et al., designed a detoxification system by 3D printed biomimetic nanocomposite structure in a hydrogel. This device allows the polyacetylene nanoparticles to sense and capture the toxins efficiently [275].

**Fig. 7.**
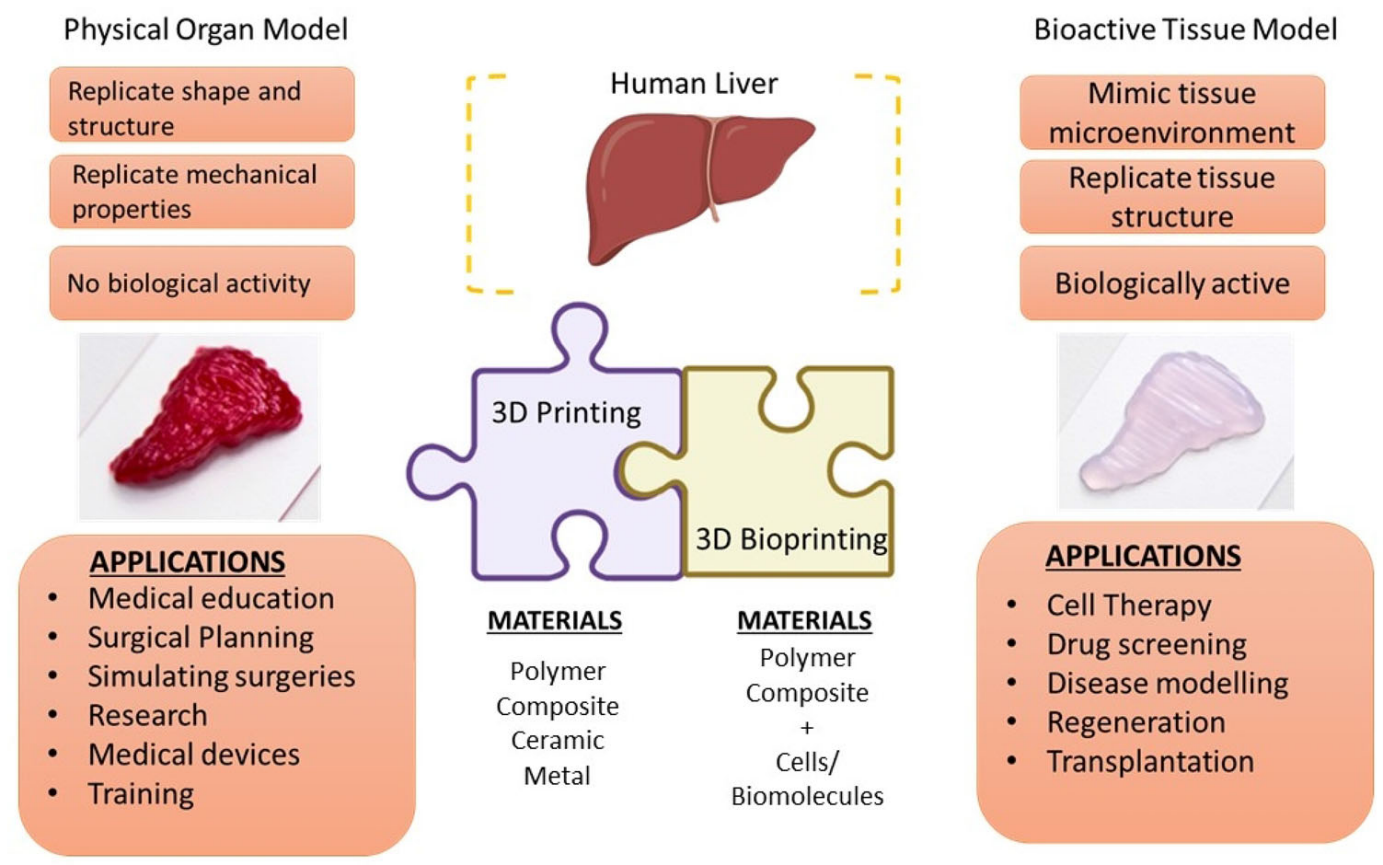
3D printing versus 3D bioprinting liver model

3D printed liver models facilitate pre-surgical planning, medical education, alternate approaches to regenerate liver tissues, evaluation of hepatotoxicity and various drugs; which makes this technology superior over other traditional methods in fabricating scaffolds and tissue models [276]. The same is illustrated in Fig. 7.

### Commercial prospects

While several research groups have made significant progress in developing functional liver tissue using 3D bioprinting, the technology is still in the early stages of development and may take several years for commercial availability of 3D bioprinted liver tissue.

Organovo, a US based company established in 2007 specialises in the design and development of 3D human tissues for research and therapeutic applications. The company synthesised human liver tissue (up to 500 microns in thickness) with their proprietary NovoGen™ bioprinter. The construct was bioprinted using human hepatocytes, liver endothelial cells, hepatic stellate cells, and HUVECs. They showed that the fabricated construct mimicked several biological functions such as albumin, fibrinogen, transferrin, and cholesterol production. They also showed that the fabricated patch can be implanted for up to 90 days in animal disease models [277–279]. Their bioprinter human liver tissue patch is termed as ExVive™. This fabricated construct was proved to be reliable for the study of complex, chronic conditions such as steatosis, fibrosis and NASH [117, 122, 280, 281]. Advanced Solutions Life Sciences, a US based company established in 1987 has launched a 3D bioprinting platform called BioAssembly Bot with its early prototype in 2013 and holds patents. BioBots, USA developed a human liver tissue using their commercially available bioink called BioGel and HepG2 cells. The BioBots demonstrated the fabrication of high-resolution liver tissue construct and it remained viable for 2 weeks [282]. Sarah et al. demonstrated the automated fabrication of a vascularized human liver tissue construct using primary human hepatocytes and non-parenchymal cells along with isolated fragments of intact human micro vessels as vascular precursors [283]. Aspect Biosystems, a Canadian company is developing allogeneic tissue therapeutics using their proprietary bioprinting technology. A therapeutic program is dedicated to restore lost or damaged liver functions. A bioprinted cell therapy using human hepatocytes is being developed and is currently at pre-clinical stage. It was demonstrated that the bioprinted tissue was viable for 28 days in mice [284, 285]. RegenHU, a Swiss company founded in 2007 specialises in 3D bioprinting technology and is currently developing a hepatic tissue for the replacement of liver transplantation for end-stage liver failure patients [286, 287]. T&R Biofab, a Korean based company established in 2013 is focused on developing bioresorbable scaffolds using 3D printing technology. The company was successful in patterning spherical tissues into structures (equivalent of liver lobes). These structures were implanted in mice and it showed good viability and structural stability. No cell death or fibrotic tissue was observed [288–291]. Cyfuse Biomedical, a Japanese company founded in 2010 develops tissue fabrication systems. Their proprietary technology “Bio 3D Printing” is a scaffold free approach to fabricating artificial organs. Spheroids are stacked on an array needle by the Bio 3D Printer and after the cells fuse, the needle is removed, leaving behind a 3D structure made with cells only. This technology was applied in developing a liver tissue for drug discovery and is currently in preclinical stage. The study demonstrated diverse liver metabolic functions for 2 weeks. The study also showed that the printed liver-maintained drug metabolic functions for 50 days [292]. Yanagi et al., reported a transplantation method for growing liver buds *in vivo*. This study also demonstrated fabrication of scalable liver tissues using Bio 3D Printer, exhibited self-organisation of tissue and successful engraftment on rats [293].

## Challenges and future directions

3D bioprinted liver models are being developed rapidly over the past few years due to the increasing need of models that can mimic the cell microenvironment accurately. Although 3D bioprinting helps simulate *in vivo* microenvironment, there is still a long way to go before arriving at a fully functional organ. This is because most research focuses on evaluating only specific liver functions and fails to analyse all the functional capabilities [47]. Another main challenge is vascularization, although few studies reported to model a vascularized liver tissue, it is still in infancy and there remains a huge gap to design improvement. The development of *in vitro* liver constructs by 3D bioprinting will improve exponentially in the coming years due to advanced ongoing research in biomaterials, bioinks, cell biology and molecular medicine.

## Conclusion

3D bioprinted *in vitro* liver structures offer promising results for effective drug screening and disease models, enhance liver regeneration and transplantation. However, its clinical translation depends on how closely the 3D bioprinted model can mimic the functions of a native liver, which requires extensive optimization in the process. 3D bioprinting allows fabrication of macroscopic and microscopic sections in an organ making it a unique technology for precise fabrication of vascular and ductular structures in a liver. This capacity to develop an organ with different cell types and biomaterials was made feasible with 3D bioprinting technology. This field so far has paved the way for research in biomaterials, bioinks, printing techniques, design strategies and a lot of methods were developed to promote cell viability and proliferation on scaffolds.

## Data Availability

The datasets used and/or analyzed during the present study are available from the corresponding author on reasonable request.
